# Supplementary material for: Endogenous hydrogen peroxide positively regulates secretion of a gut-derived peptide in neuroendocrine potentiation of the oxidative stress response in C. elegans
Source: bioRxiv. 2024 Sep 22:2024.04.03.587937. Preprint. [Version 2] doi: 10.1101/2024.04.03.587937 (PMC11429608; doi:10.1101/2024.04.03.587937)
Supplement: Supplement 1 [file NIHPP2024.04.03.587937v2-supplement-1.pdf]

bioRxiv preprint doi: https://doi.org/10.1101/2024.04.03.587937; this version posted September 22, 2024. The copyright holder for this preprint (which was not certified by peer review) is the author/funder, who has granted bioRxiv a license to display the preprint in perpetuity. It is made available under aCC-BY 4.0 International license.

Supplementary Figures

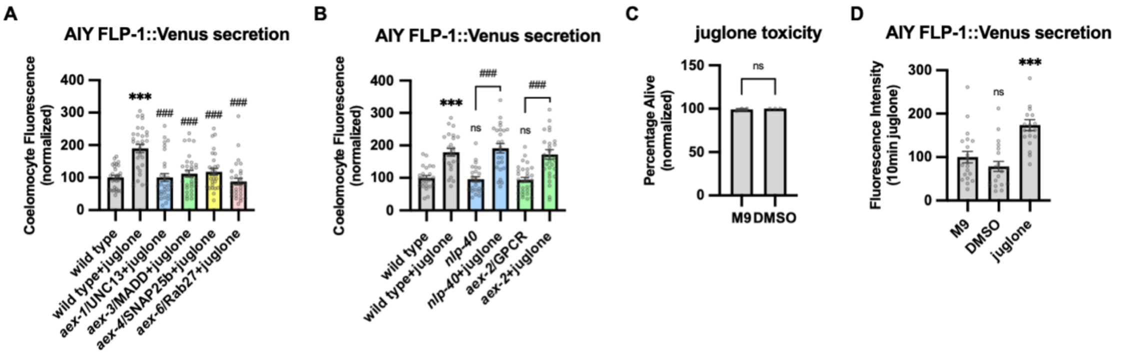

Supplementary Figure 1. The effect of intestinal DCV secretion mutations on FLP-1 release from AIY.
A Quantification of average coelomocyte fluorescence of the indicated mutants expressing FLP-1::Venus fusion proteins in AIY following M9 or 300μM juglone treatment for 10min. Unlined *** and ### denotes statistical significance compared to “wild type”. n = 30, 30, 29, 30, 30, 30 independent animals.
B Quantification of average coelomocyte fluorescence of the indicated mutants expressing FLP-1::Venus fusion proteins in AIY following M9 or 300μM juglone treatment for 10min. Unlined *** and ns denote statistical analysis compared to “wild type”. n = 24, 24, 25, 25, 30, 30 independent animals.
C Average percentage of surviving young adult animals of the indicated genotypes after 16h recovery following 4h DMSO treatment. n = 203, 174 independent biological samples over three independent experiments.
D Quantification of average coelomocyte fluorescence of the indicated mutants expressing FLP-1::Venus fusion proteins in AIY following M9, DMSO or juglone treatment for 10min. Unlined ns and *** denote statistical significance compared to “M9”. n = 20, 20, 19 independent animals.
A-D Data are mean values ± s.e.m normalized to wild type controls. **A, B and D** ns. not significant, *** and ### *P* < 0.001 by Brown-Forsythe and Welch ANOVA with Dunnett’s T3 multiple comparisons test. **C** ns. not significant by unpaired t test with Welch’s correction.

bioRxiv preprint doi: https://doi.org/10.1101/2024.04.03.587937; this version posted September 22, 2024. The copyright holder for this preprint (which was not certified by peer review) is the author/funder, who has granted bioRxiv a license to display the preprint in perpetuity. It is made available under aCC-BY 4.0 International license.

Supplementary Figure 2. Specificity of juglone on intestinal peptide secretion, and FLP-2 and NLP-40 localization in the intestine.

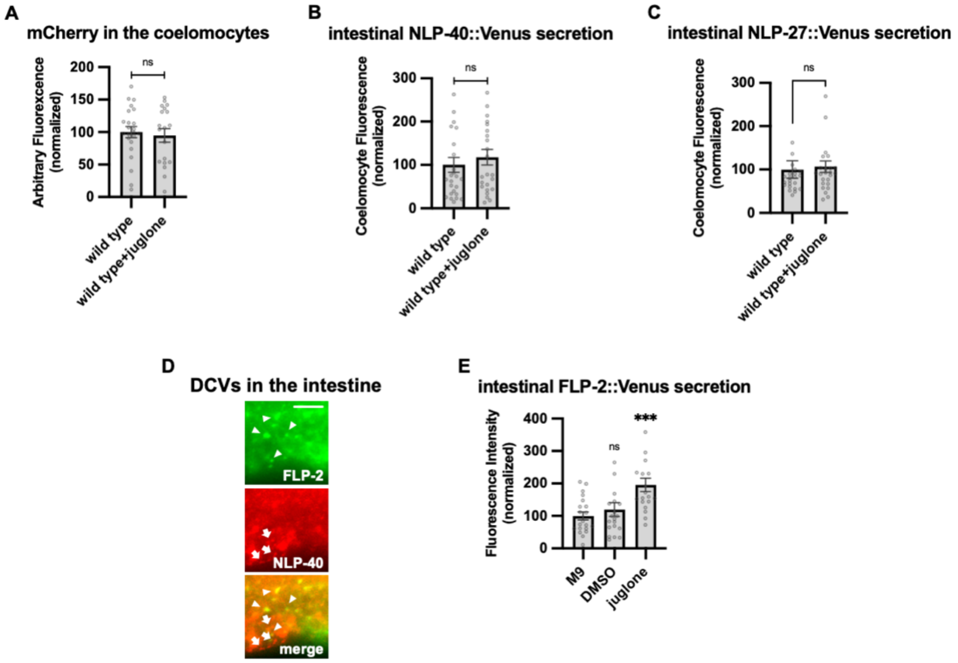

A Quantification of average coelomocyte fluorescence of the indicated mutants co-expressing FLP-2::Venus in the intestine (under the *ges-1* promoter) and mCherry in the coelomocytes (under the *ofm-1* promoter) following M9 or 300μM juglone treatment for 10min. n = 23, 19 independent animals.
B Quantification of average coelomocyte fluorescence of transgenic animals expressing NLP-40::Venus fusion proteins in the intestine following M9 or 300μM juglone exposure for 10min. n = 25, 24 independent animals.
C Quantification of average coelomocyte fluorescence of transgenic animals expressing NLP-27::Venus fusion proteins in the intestine following M9 or 300μM juglone exposure for 10min. n = 23, 25 independent animals.
D Representative images of fluorescence distribution in the posterior intestinal region of transgenic animals co-expressing FLP-2::Venus fusion proteins (marked by arrowheads) and NLP-40::mTur2 fusion proteins (marked by arrows). Scale bar: 5μM.
E Quantification of average coelomocyte fluorescence of transgenic animals expressing FLP-2::Venus fusion proteins in the intestine following M9, DMSO or 300μM juglone exposure for 10min. Unlined ns and *** denote statistical significance compared to "M9". n = 20, 20, 20 independent animals.
A-C and E Data are mean values ± s.e.m normalized to wild type controls. **A-C** ns. not significant by unpaired t test with Welch's correction. **E** ns. not significant by Brown-Forsythe and Welch ANOVA with Dunnett's T3 multiple comparisons test.

bioRxiv preprint doi: https://doi.org/10.1101/2024.04.03.587937; this version posted September 22, 2024. The copyright holder for this preprint (which was not certified by peer review) is the author/funder, who has granted bioRxiv a license to display the preprint in perpetuity. It is made available under aCC-BY 4.0 International license.

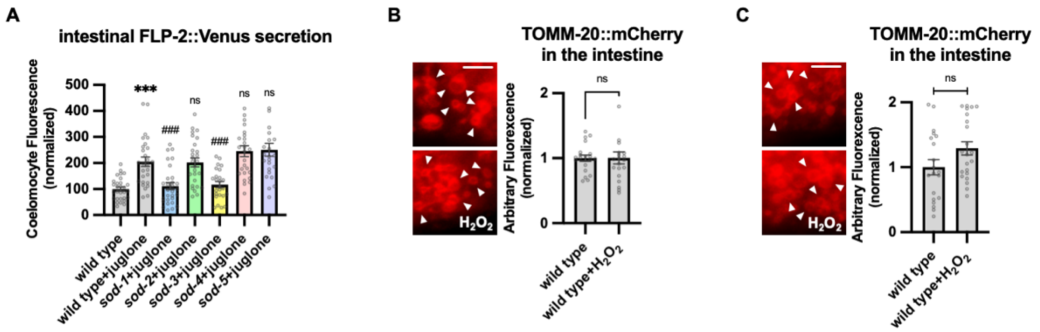

Supplementary Figure 3. SODs function in juglone induced FLP-2 release from the intestine and mitochondrial mCherry control.

A Quantification of average coelomocyte fluorescence of the indicated mutants expressing FLP-2::Venus fusion proteins in the intestine following M9 or juglone treatment for 10min. Unlined *** denotes statistical significance compared to “wild type”; unlined ### and ns denote statistical significance compared to “wild type + juglone” n = 29, 27, 29, 27, 25, 26, 24 independent animals.

B and C Representative images and quantification of average fluorescence intensity of TOMM-20::mCherry proteins in transgenic animals co-expressing matrix-HyPer7 (B) or OMM-HyPer7 (C) following M9 or H_2O_2 treatment for 10min. (B) Scale bar: Scale bar: 5μM. n = 20, 20 independent animals. (C) Scale bar: Scale bar: 5μM. n = 20, 22 independent animals.

A-C Data are mean values ± s.e.m normalized to wild type controls. **A** ns. not significant, *** and ### $P < 0.001$ by Brown-Forsythe and Welch ANOVA with Dunnett’s T3 multiple comparisons test. **B and C** ns. not significant by unpaired t test with Welch’s correction.

bioRxiv preprint doi: https://doi.org/10.1101/2024.04.03.587937; this version posted September 22, 2024. The copyright holder for this preprint (which was not certified by peer review) is the author/funder, who has granted bioRxiv a license to display the preprint in perpetuity. It is made available under aCC-BY 4.0 International license.

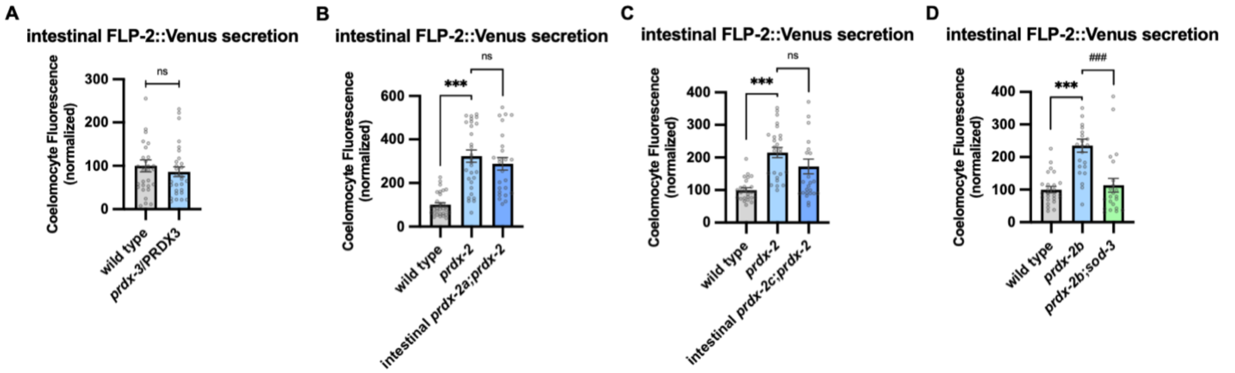

Supplementary Figure 4. PRDX-2 intestinal rescue and mediates SOD-3 dependent regulation of FLP-2 release.

A Quantification of average coelomocyte fluorescence of the indicated mutants expressing FLP-2::Venus fusion proteins in the intestine following M9 treatment for 10min. n = 30, 29 independent animals.

B Quantification of average coelomocyte fluorescence of the indicated mutants expressing FLP-2::Venus fusion proteins in the intestine following M9 treatment for 10min. Intestinal *prdx-2a* denotes expression of *prdx-2a* cDNA under the *ges-1* promoter. n = 30, 30, 25 independent animals.

C Quantification of average coelomocyte fluorescence of the indicated mutants expressing FLP-2::Venus fusion proteins in the intestine following M9 treatment for 10min. Intestinal *prdx-2c* denotes expression of *prdx-2c* cDNA under the *ges-1* promoter. n = 25, 25, 25 independent animals.

D Quantification of average coelomocyte fluorescence of the indicated mutants expressing FLP-2::Venus fusion proteins in the intestine following M9 treatment for 10min. n = 25, 23, 22 independent animals.

A-D Data are mean values ± s.e.m normalized to wild type controls. **A** ns. not significant by unpaired t test with Welch's correction. **B-D** ns. not significant, *** and ### $P < 0.001$ by Brown-Forsythe and Welch ANOVA with Dunnett's T3 multiple comparisons test.

bioRxiv preprint doi: https://doi.org/10.1101/2024.04.03.587937; this version posted September 22, 2024. The copyright holder for this preprint (which was not certified by peer review) is the author/funder, who has granted bioRxiv a license to display the preprint in perpetuity. It is made available under aCC-BY 4.0 International license.

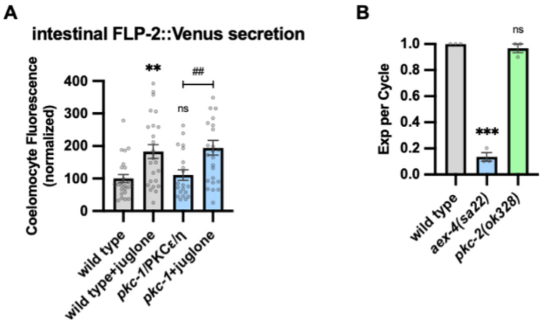

Supplementary Figure 5. Juglone promotes FLP-2 release in *pkc-1* mutants and expulsion analysis.
A Quantification of average coelomocyte fluorescence of the indicated mutants expressing FLP-2::Venus fusion proteins in the intestine following M9 or juglone treatment for 10min. Unlined ns and ** denote statistical significance compared to “wild type”. n = 24, 25, 20, 25 independent animals.
B Quantification of the number of expulsions (Exp) per defecation cycle in adult animals of the indicated genotypes. Unlined *** and ns denote statistical significance compared to “wild type”. n = 30, 30, 30, 30 in three independent animals.
A-B Data are mean values ± s.e.m normalized to wild type controls. ns. not significant, ** and ## $P < 0.01$, *** $P < 0.001$ by Brown-Forsythe and Welch ANOVA with Dunnett’s T3 multiple comparisons test.

bioRxiv preprint doi: https://doi.org/10.1101/2024.04.03.587937; this version posted September 22, 2024. The copyright holder for this preprint (which was not certified by peer review) is the author/funder, who has granted bioRxiv a license to display the preprint in perpetuity. It is made available under aCC-BY 4.0 International license.

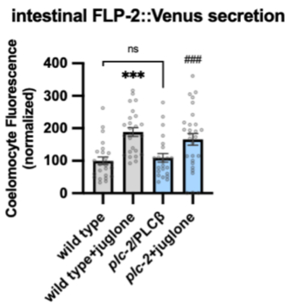

Supplementary Figure 6. Juglone promotes FLP-2 release in *plc-2* mutants.
Quantification of average coelomocyte fluorescence of the indicated mutants expressing FLP-2::Venus fusion proteins in the intestine following M9 or juglone treatment for 10min. Unlined *** denotes statistical significance compared to “wild type”; unlined ### denotes statistical significance compared to “*plc-2*/PLCβ”. n = 25, 25, 23, 28 independent animals.
Data are mean values ± s.e.m normalized to wild type controls. ns. not significant, *** and ### $P < 0.001$ by Brown-Forsythe and Welch ANOVA with Dunnett’s T3 multiple comparisons test.

bioRxiv preprint doi: https://doi.org/10.1101/2024.04.03.587937; this version posted September 22, 2024. The copyright holder for this preprint (which was not certified by peer review) is the author/funder, who has granted bioRxiv a license to display the preprint in perpetuity. It is made available under aCC-BY 4.0 International license.

1478 **Table S1. Strains, transgenic lines and plasmids used in this study**

Strain	Genotype	Figures
N2	wild type Bristol strain	Fig. 1C, S1C, S5B
OJ6555	*flp-1(ok2811) IV*	Fig. 1C
OJ5490	*flp-2(ok3351) X*	Fig. 1C
OJ10228	*flp-1(ok2811);flp-2(ok3351)*	Fig. 1C
OJ7466	*aex-4(sa22) X*	Fig. S5B
VC127	*pkc-2(ok328) X*	Fig. S5B
OJ3614	*vjIs150[pJQ60(Pttx-3::flp-1::Venus)]*	Fig. 1A, 1B, 1D, S1B, S1D
OJ5616	*aex-5(sa23);vjIs150*	Fig. 1B
OJ5780	*vjEx1748[pJQ298(Prab-3::aex-5 cDNA)];aex-5(sa23);vjIs150*	Fig. 1B
OJ5785	*vjEx1753[pJQ299(Pges-1::aex-5 cDNA)];aex-5(sa23);vjIs150*	Fig. 1B
OJ6334	*vjEx1753;aex-5(sa23);flp-2(ok3351);vjIs150*	Fig. 1B
OJ5264	*flp-2(ok3351);vjIs150*	Fig. 1D
OJ8818	*vjEx2882[pJQ366(Prab-3::flp-2 gDNA)];flp-2(ok3351);vjIs150*	Fig. 1D
OJ8813	*vjEx2877[pJQ305(ges-1::flp-2 gDNA)];flp-2(ok3511);vjIs150*	Fig. 1D
OJ10229	*vjEx2877;vjIs150*	Fig. 1D
CL2166	*dvIs19[pAF15(Pgst-4::GFP::NLS)]*	Fig. 1F, 1G, 4F
OJ2547	*flp-1(ok2811);dvIs19*	Fig. 1F, 1G
OJ10230	*flp-2(ok3351);dvIs19*	Fig. 1F
OJ6544	*flp-1(ok2811);flp-2(ok3511);dvIs19*	Fig. 1F
OJ10231	*vjEx2877;dvIs19*	Fig. 1G
OJ10232	*vjEx2877;flp-1(ok2281);dvIs19*	Fig. 1G
OJ5888	*aex-1(sa9);vjIs150*	Fig. S1B
OJ5890	*aex-3(js815);vjIs150*	Fig. S1B
OJ5891	*aex-4(sa22);vIs150*	Fig. S1B
OJ5892	*aex-6(sa24);vjIs150*	Fig. S1B
OJ5615	*nlp-40(tm4085);vjIs150*	Fig. S1C
OJ5889	*aex-2(sa3);vjIs150*	Fig. S1C
OJ6405	*vjEx2035[pJQ305(Pges-1::flp-2 gDNA::Venus)]*	Fig. 2A, 2C, 2D, 2F, S2A, S2E, 3A, 3B, 3C, 3G, 3H, S3A, 4B, 4E, S4A, S4B, S4C, S4D, 5A, 5D, 5E, S5A, 6B, 6E, 6H, S6
OJ9469	*vjEx3069[pDY10(Pges-1::aex-5::mTur2)];vjEx2035*	Fig. 2B
OJ6409	*aex-4(sa22);vjEx2035*	Fig. 2C, 3G
OJ8345	*aex-6(sa24);vjEx2035*	Fig. 2C, 3G
OJ6641	*flp-1(ok2811);vjEx2035*	Fig. 2F
OJ1002	*vjIs40[pDS292(Pnlp-40::nlp-40::Venus)]*	Fig. S2B
OJ10237	*vjEx3263[pJQ370(Pges-1::nlp-27 gDNA::Venus)]*	Fig. S2C
OJ9567	*vjEx3062[pDY(Pges-1::nlp-40::mTur2)];vjEx2035*	Fig. S2D
OJ9797	*sod-1(tm783);vjEx2035*	Fig. 3A, 3H, S3A
OJ8588	*vjEx2814[pJQ419(Pges-1::sod-1b cDNA)];sod-1(tm783);vjEx2035*	Fig. 3A
OJ8341	*sod-3(tm760);vjEx0235*	Fig. 3B, 3H, S3A
OJ8933	*vjEx2910[pJQ389(Pges-1::sod-3 cDNA)];sod-3(tm760);vjEx2035*	Fig. 3B
OJ9106	*vjEx2973[pJQ408(Pges-1::sod-3(ΔMLS) cDNA)];sod-3(tm760);vjEx2035*	Fig. 3B
OJ10234	*sod-1(tm783);sod-3(tm760);vjEx2035*	Fig. 3C, 3H
OJ10243	*vjEx3266[pJQ420(Pges-1::sod-1b cDNA::GFP)]*	Fig. 3D
OJ9141	*vjEx2993[pJQ407(Pges-1::sod-3 cDNA::GFP)]*	Fig. 3E
OJ9144	*vjEx2996[pJQ409(Pges-1::sod-3(ΔMLS) cDNA::GFP)]*	Fig. 3F

bioRxiv preprint doi: https://doi.org/10.1101/2024.04.03.587937; this version posted September 22, 2024. The copyright holder for this preprint (which was not certified by peer review) is the author/funder, who has granted bioRxiv a license to display the preprint in perpetuity. It is made available under aCC-BY 4.0 International license.

OJ9230	*vjEx3020[pJQ383(Pges-1::MLS::HyPer7)]*	Fig. 3I, S3B, 5B, 6C, 6F
OJ9196	*vjEx3014[pJQ411(Pges-1::tomm-20::HyPer7)]*	Fig. 3I, S3C, 5C ,6D, 6G
OJ9281	*sod-1(tm783);vjEx3020*	Fig. 3I
OJ9259	*sod-3(tm760);vjEx3020*	Fig. 3I
OJ10244	*sod-1(tm783);sod-3(tm760);vjEx3020*	Fig. 3I
OJ9795	*sod-1(tm783);vjEx3014*	Fig. 3I
OJ9280	*sod-3(tm760);vjEx3014*	Fig. 3I
OJ10245	*sod-1(tm783);sod-3(tm760);vjEx3014*	Fig. 3I
OJ10238	*sod-2(ok1030);vjEx2035*	Fig. S3A
OJ10239	*sod-4(gk101);vjEx2035*	Fig. S3A
OJ10240	*sod-5(tm1146);vjEx2035*	Fig. S3A
OJ8991	*prdx-2(gk169);vjEx2035*	Fig. 4B, S3E, S3F ,4C, 5E
OJ10251	*prdx-2b(vj380);vjEx2035*	Fig. 4B, 4E, S4B
OJ8996	*vjEx2926[pJQ381(Pges-1::prdx-2b cDNA)];prdx-2(gk169);vjEx2035*	Fig. 4B
OJ9249	*trx-3(tm2820);vjEx2035*	Fig. 4B
OJ9496	*vjEx3091[pJQ422(Pges-1::trx-3 cDNA)];trx-3(tm2820);vjEx2035*	Fig. 4B
OJ10252	*trx-3(tm2820);sod-1(tm783);vjEx2035*	Fig. 4B
OJ10253	*trx-3(tm2820);sod-3(tm760);vjEx2035*	Fig. 4B
OJ9237	*prdx-2(gk169);vjEx3020*	Fig. 4C
OJ10247	*prdx-2b(vj380);vjEx3020*	Fig. 4C
OJ10249	*trx-3(tm2820);vjEx3020*	Fig. 4C
OJ10246	*prdx-2(gk169);vjEx3014*	Fig. 4D
OJ10248	*prdx-2b(vj380);vjEx3014*	Fig. 4D
OJ10250	*trx-3(tm2820);vjEx3014*	Fig. 4D
OJ10254	*prdx-2b(vj380);dvIs19*	Fig. 4F
OJ10255	*prdx-2b(vj380);flp-2(tm3351);dvIs19*	Fig. 4F
OJ10256	*prdx-3(gk529);vjEx2035*	Fig. S4A
OJ10258	*vjEx3268[pJQ380(Pges-1::prdx-2a cDNA)];prdx-2(gk169);vjEx2035*	Fig. S4B
OJ10260	*vjEx3270[pJQ399(Pges-1::prdx-2c cDNA)];prdx-2(gk169);vjEx2035*	Fig. S4C
OJ9250	*prdx-2b(vj380);sod-3(tm760);vjEx2035*	Fig. S4D
OJ9682	*pkc-2(ok328);vjEx2035*	Fig. 5A, 5D, 5E
OJ8682	*vjEx2828[pJQ376(Pges-1::pkc-2b cDNA)];pkc-2(ok328);vjEx2035*	Fig. 5A
OJ9657	*vjEx3131[pJQ446(Pges-1::pkc-2(K375R) cDNA)];pkc-2(ok328);vjEx2035*	Fig. 5A
OJ10279	*pkc-2(ok328);vjEx3020*	Fig. 5B
OJ10280	*pkc-2(ok328);vjEx3014*	Fig. 5B
OJ8939	*prdx-2(gk169);pkc-2(ok328);vjEx2035*	Fig. 5E
OJ10278	*pkc-1(nj3);vjEx2035*	Fig. S5A
OJ9863	*egl-8(sa47);vjEx2035*	Fig. 6B
OJ10281	*egl-8(sa47);vjEx3020*	Fig. 6C
OJ10282	*egl-8(sa47);vjEx3014*	Fig. 6D
OJ10263	*dgk-2(gk124);vjEx2035*	Fig. 6E, 6H
OJ10264	*vjEx327[pJQ460(Pges-1::dgk-2a cDNA)];dgk-2(gk124);vjEx2035*	Fig. 6E
OJ10266	*dgk-2(gk124);pkc-2(ok328);vjEx2035*	Fig. 6E
OJ10283	*dgk-2(gk124);vjEx3020*	Fig. 6F
OJ10284	*dgk-2(gk124);vjEx3014*	Fig. 6G
OJ9809	*plc-2(ok1761);vjEx2035*	Fig. S6
OJ9028	*vjEx2936[pJQ382(Pttx-3::MLS::HyPer7)]*	Fig. 1E

bioRxiv preprint doi: https://doi.org/10.1101/2024.04.03.587937; this version posted September 22, 2024. The copyright holder for this preprint (which was not certified by peer review) is the author/funder, who has granted bioRxiv a license to display the preprint in perpetuity. It is made available under aCC-BY 4.0 International license.

OJ10595	*vjEx2936;flp-2(ok3351)*	Fig. 1E
